# Multiple Cutaneous Leiomyomas of the Shoulder: Investigating Chronic Pain and Reed’s Syndrome Association

**DOI:** 10.7759/cureus.82070

**Published:** 2025-04-11

**Authors:** Moiz Alam, Samujjala Deb, Mahak Sharma, Sarah Lateef

**Affiliations:** 1 Dermatology, New York Institute of Technology College of Osteopathic Medicine, New York, USA; 2 Dermatology, Inskin Clinic, Durgapur, IND

**Keywords:** cigar-shaped nuclei, cutaneous leiomyoma, painful nodules, reed’s syndrome, renal cell carcinoma (rcc)

## Abstract

Cutaneous leiomyomas are rare benign tumors originating from the arrector pili muscles, often presenting as firm, painful nodules. In this report, we describe a man in his 40s presenting with multiple progressively enlarging painful nodules on the right shoulder first noticed about five years ago. The pain was described to be paroxysmal, worsened by cold exposure and physical pressure, and described as burning and aching. A 4-mm punch biopsy confirmed the diagnosis of multiple cutaneous leiomyomas, showing fascicles of spindle cells and elongated cigar-shaped nuclei in a palisading pattern. The patient was treated with oral nifedipine for pain control, with topical hyoscine hydrobromide (9%) recommended if symptoms persisted. He was advised to follow up for worsening pain and screening for potential Reed’s syndrome. Here, we emphasize the importance of considering cutaneous leiomyoma in the differential diagnosis of painful dermal nodules and the significance of evaluating for possible syndromic associations.

## Introduction

Leiomyomas are benign smooth muscle tumors that can arise in various tissues, most commonly in the uterus, skin, and gastrointestinal tract. These tumors are typically well-circumscribed, slow-growing, and hormonally independent, except for uterine leiomyomas, which are estrogen-dependent. Among the different types, uterine leiomyomas (fibroids) are the most prevalent, affecting up to 70-80% of women by age 50, often leading to abnormal uterine bleeding, infertility, and pelvic pain [1,2]. Gastrointestinal leiomyomas, although less common, typically arise in the esophagus, stomach, or intestines and are often discovered incidentally unless they cause obstruction, bleeding, or perforation. Vascular leiomyomas (angioleiomyomas) originate from vascular smooth muscle, frequently occurring in the lower extremities, where they are often painful, particularly in response to cold or pressure. Among these subtypes, cutaneous leiomyomas are a distinct entity that arise specifically from the arrector pili muscles of the skin. Cutaneous leiomyomas, also known as piloleiomyomas, are rare benign neoplasms derived from smooth muscle tissue in the skin. They typically present as firm, painful, skin-colored nodules, either solitary or multiple. Solitary cutaneous leiomyomas are sporadic lesions that usually develop in young to middle-aged adults. They range in size from 0.5 to 2 cm and are often misdiagnosed as dermatofibromas or neurofibromas due to their overlapping clinical features, leading to diagnostic delays. Multiple piloleiomyomas, on the other hand, are frequently associated with Reed’s syndrome. This autosomal dominant disorder, caused by mutations in the fumarate hydratase (FH) tumor suppressor gene, increases the risk of type 2 papillary renal cell carcinoma [2]. Patients with Reed’s syndrome often develop clusters of cutaneous leiomyomas, particularly on the trunk and extremities, and women with the syndrome frequently have uterine fibroids. 

The management of cutaneous leiomyomas primarily focuses on symptom relief, with surgical excision typically reserved for isolated, symptomatic lesions. While a single leiomyoma can often be surgically removed with complete symptom resolution, patients with multiple or widespread lesions generally require medical therapy as the first-line approach. Several pharmacologic options have been explored for pain management. Calcium channel blockers, such as nifedipine, diltiazem, and verapamil, help reduce pain by blocking calcium ion influx into smooth muscle cells, thereby preventing painful contractions [2]. Alpha-adrenergic blockers, like phenoxybenzamine, have also shown efficacy in controlling paroxysmal pain by inhibiting smooth muscle contraction and nerve stimulation. In addition, some studies have reported partial symptom relief with topical nitroglycerin, though its effectiveness remains inconsistent. In cases of refractory pain, localized botulinum toxin (Botox) injections have been explored as an alternative therapy to reduce muscle contractions and alleviate discomfort [2]. 

This case report aims to contribute to the limited clinical literature on multiple cutaneous leiomyomas by presenting a localized and symptomatic case involving the shoulder, emphasizing diagnostic challenges, the importance of evaluating for syndromic associations such as Reed’s syndrome, and the role of conservative medical management.

## Case presentation

**Figure 1:**
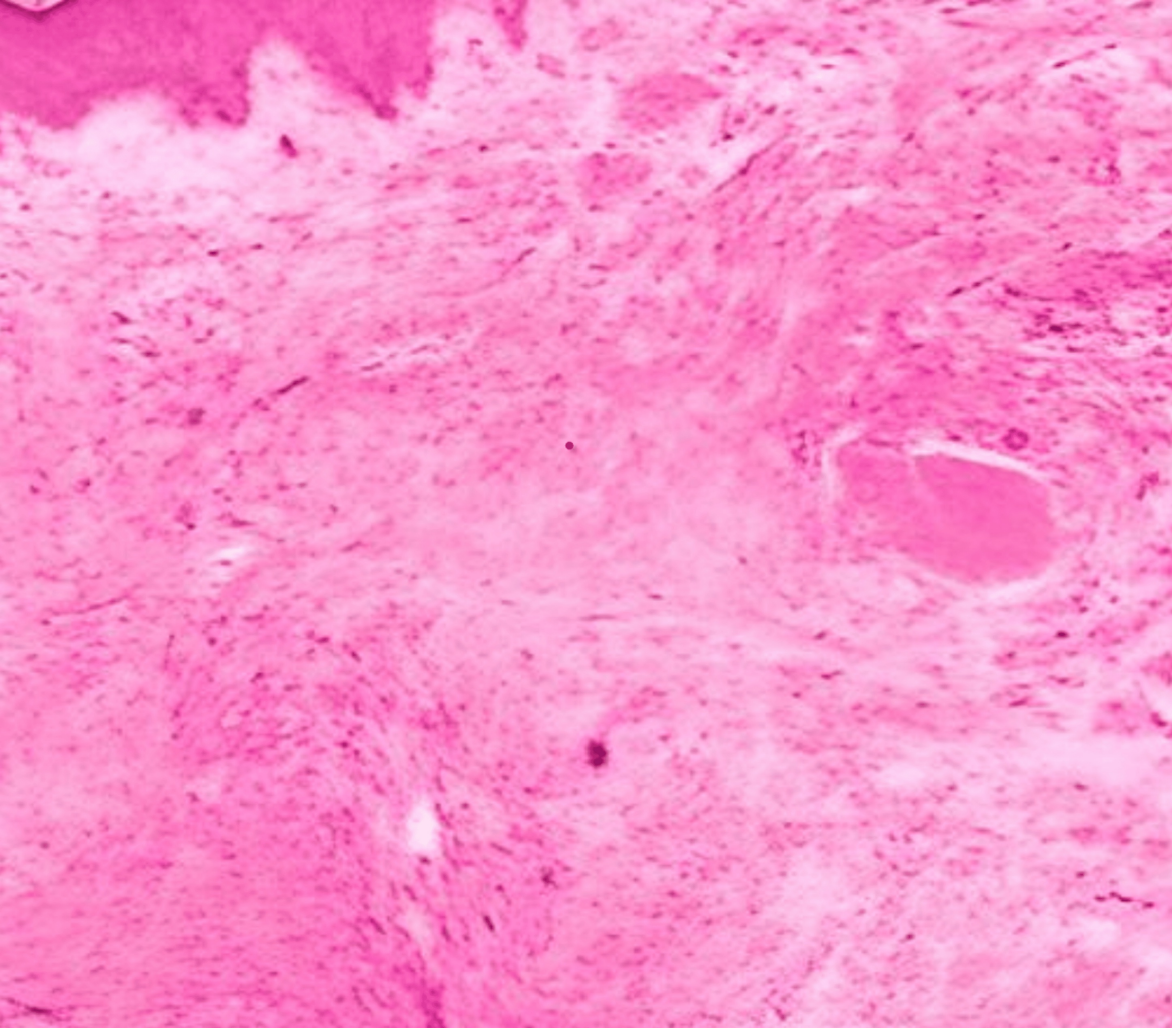
Histopathology demonstrating interlacing fascicles of spindle cells with abundant eosinophilic cytoplasm and elongated, cigar-shaped nuclei arranged in a palisading pattern within the dermis.

A man in his 40s presented to the outpatient clinic with multiple small, firm nodules on his right shoulder first noticed approximately five years ago (Figure 1). Initially asymptomatic, these nodules gradually increased in size and number over time. About three years after their onset, the patient began experiencing intermittent burning and aching pain, which progressively worsened over the following two years. The pain was paroxysmal, exacerbated by cold exposure and physical pressure, and became significantly disruptive to his daily activities. Despite the increasing discomfort, the patient did not seek medical attention until two years after symptom onset, when the pain became persistent and difficult to manage. 

**Figure 2:**
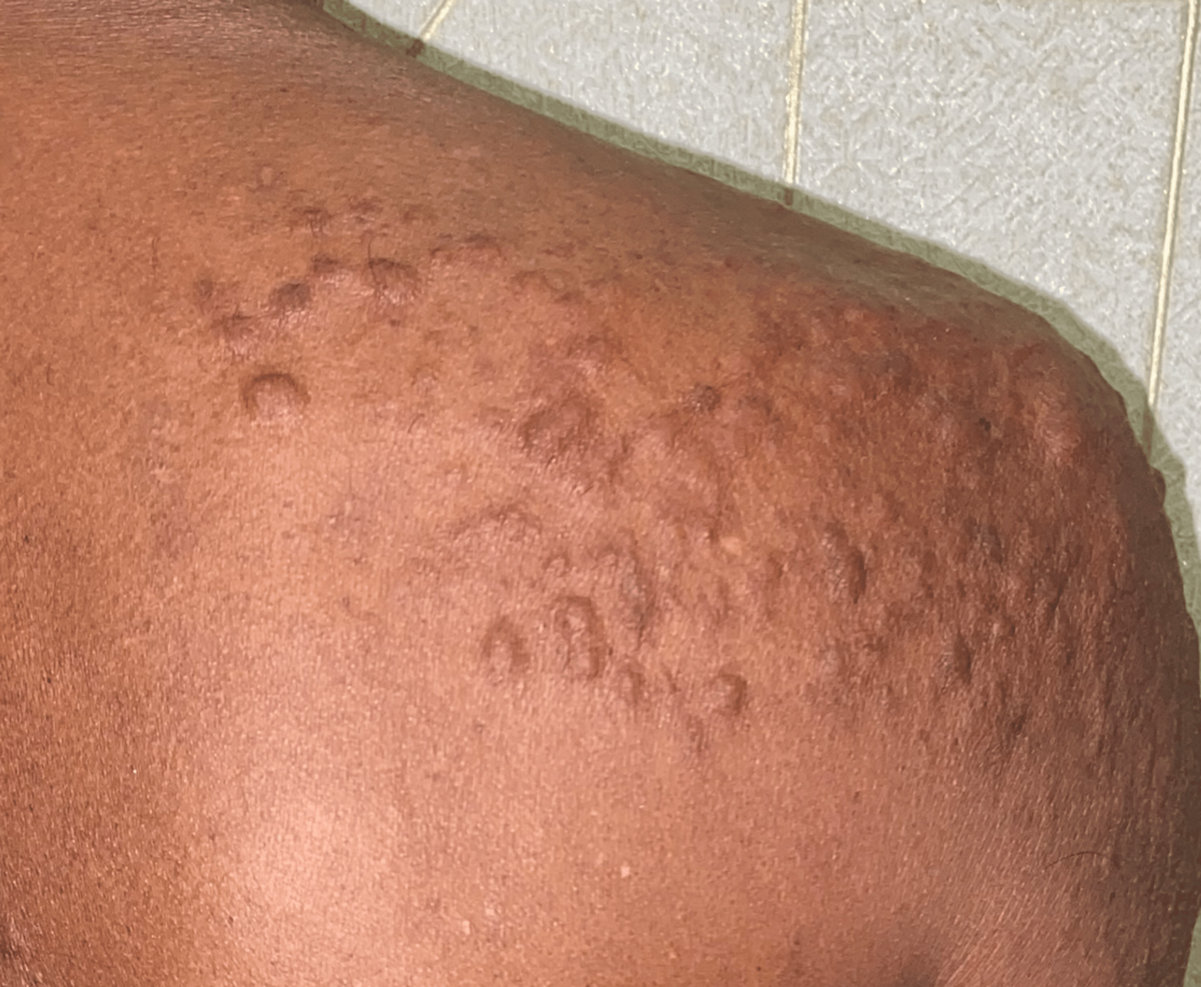
Multiple well-defined, dome-shaped, skin-colored nodules clustered over the right shoulder, consistent with the clinical appearance of cutaneous leiomyomas.

Upon his initial clinical evaluation, the patient denied any history of itching, discharge, or systemic symptoms such as fever, weight loss, night sweats, or malaise. He had no previous dermatological conditions and no significant past medical history, including no prior diagnosis of hypertension, diabetes, malignancy, or connective tissue disorders. There was no family history of similar skin lesions, leiomyomas, uterine fibroids, or renal cell carcinoma. His lifestyle history revealed that he was a non-smoker, consumed alcohol occasionally, and had no history of illicit drug use. He worked in an office-based job with a sedentary lifestyle. On physical examination, multiple discrete, dome-shaped, skin-colored to slightly erythematous nodules were present on the right shoulder, ranging in size from 0.5 cm to 2 cm (Figure 2). On palpation, the nodules were firm, mobile, and tender to touch, particularly when pressure was applied. In addition, the nodules were non-ulcerated, with no erythema, scaling, or discharge. They were freely mobile under the skin and exhibited no surface changes or signs of secondary infection. There were no abnormal findings in other areas of the skin, mucous membranes, or nails. His vital signs were stable, with no evidence of systemic involvement. Given the presence of multiple lesions, further evaluation for Reed’s Syndrome was recommended to assess the risk of hereditary leiomyomatosis and renal cell carcinoma (HLRCC). 

A 4-mm punch biopsy was performed under local anesthesia to establish a definitive diagnosis. Histopathological examination revealed interwoven fascicles of spindle cells with abundant eosinophilic cytoplasm and elongated cigar-shaped nuclei arranged in a palisading pattern within the dermis, consistent with cutaneous leiomyoma (Figure 1).

There were no signs of mitotic activity, cellular atypia, or malignancy, effectively ruling out leiomyosarcoma or other malignant neoplasms. These findings confirmed the benign nature of the lesions and supported the diagnosis of cutaneous leiomyoma.

Following histopathological confirmation of multiple cutaneous leiomyomas, the patient was started on oral nifedipine (10 mg three times daily) for pain management, with topical hyoscine hydrobromide (9%) prescribed as adjunctive therapy if symptoms persisted. Due to the multiple lesions, surgical excision was not considered, and the treatment plan focused on long-term symptom control and monitoring. The patient was advised to track any worsening pain, changes in lesion size, or the appearance of new lesions and to return for regular follow-up evaluations. At his first follow-up visit, one month later, the patient reported mild improvement in pain intensity, though he continued to experience episodic discomfort, particularly in colder environments. He did not develop any new lesions, and there were no overt signs of renal dysfunction. However, given the presence of multiple cutaneous leiomyomas, he remained under ongoing surveillance for Reed’s Syndrome, an autosomal dominant condition associated with renal cell carcinoma.

## Discussion

Cutaneous leiomyomas, particularly in their multiple form, present significant diagnostic and therapeutic challenges due to their variable presentation and chronic pain. Differential diagnosis is a key step in the evaluation of dermal nodules, as cutaneous leiomyomas can clinically mimic several benign lesions. The most common mimickers include dermatofibromas, which are usually firm, hyperpigmented, and dimple on compression; neurofibromas, which tend to be softer and may exhibit the buttonhole sign; and epidermoid cysts, which are often central, dome-shaped, and mobile with possible punctum. Unlike these, leiomyomas are tender or painful, particularly in response to cold or pressure, and do not usually present with secondary skin changes or discharge. Histopathological evaluation remains the gold standard to distinguish these conditions definitively. The management of cutaneous leiomyomas ranges from definitive surgical excision to pharmacologic and non-invasive therapies, depending on the number of lesions, severity of symptoms, and the presence of syndromic associations. A tailored, patient-centered approach remains essential. For solitary leiomyomas, surgical excision is the definitive treatment and often results in complete symptom resolution. In some cases, skin grafting may follow to optimize cosmetic outcomes [1]. However, in patients with multiple lesions, such as those associated with Reed’s syndrome or hereditary leiomyomatosis and renal cell carcinoma (HLRCC), surgical management becomes more complex. The procedure is often limited by extensiveness, cosmetic concerns, and a high recurrence rate of up to 50%, occurring anywhere from weeks to years after excision [2,3]. In such cases, medical management becomes the preferred approach. Calcium channel blockers, particularly nifedipine, have shown efficacy in reducing pain by inhibiting calcium influx into smooth muscle cells, thereby preventing painful contractions [4]. Although not curative, nifedipine can significantly improve patient comfort. Other pharmacologic agents, including gabapentin and phenoxybenzamine, have also demonstrated pain-relieving properties by targeting neuropathic pathways or smooth muscle tone, respectively. However, the evidence supporting their sole use remains limited [4]. Since medications do not eliminate the underlying tumor burden, alternative therapies are also considered in patients with multiple, painful lesions or those unfit for surgery. Botulinum toxin A injections have been shown to inhibit the release of neuropeptides and reduce central and peripheral pain signaling, thereby decreasing the frequency and severity of painful episodes and reducing the need for systemic medication [5,6]. Cryotherapy, which uses liquid nitrogen to destroy dermal nerve fibers, has been effective in interrupting pain transmission in localized lesions [7]. Similarly, CO₂ laser ablation offers the dual benefit of cosmetic refinement and symptom control by ablating superficial tissue and nerve endings, making it a non-invasive alternative to surgical excision in selected cases [8]. It is important to note that while surgical excision is curative, it is often impractical in patients with widespread or recurrent disease. Therefore, long-term pain management, rather than lesion eradication, becomes the primary goal for these individuals.

In a histopathological context, cutaneous leiomyomas present as intertwining fascicles of spindle cells with cigar-shaped nuclei, organized in a spiral-like pattern [9].Impulsive muscle contractions and nerve entanglement within the fibrous tumor tissue are suggested to be associated with the pain arising from these lesions. When muscle fibers are exposed to external stimuli - such as cold and pressure - paroxysmal pain is experienced by the patient [10]. In Reed’s syndrome, fumarate hydratase gene mutations are associated with dysfunctional cellular metabolism and subsequent buildup of fumarate [11]. This can cause tumorigenesis by activating hypoxia inducible factors and increasing oxidative stress [12]. According to dermatologic guidelines, histopathological examination is the most updated method of preference for diagnosing cutaneous leiomyomas. To rule out other differential diagnoses such as dermatofibroma or epidermal cysts, a 4 mm punch biopsy is performed to examine the presence of smooth muscle augmentation [13]. If multiple tumors are seen, genetic testing for FH mutations is recommended in order to rule out Reed’s syndrome. This is due to its potential to manifest as hereditary leiomyomatosis and renal cell carcinoma in the patient. Patients can also be advised to go under magnetic resonance imaging or ultrasounds to determine whether renal abnormalities are present. This case examines the effect of delayed diagnosis due to slow symptomatic progression and encourages increased clinical education on cutaneous leiomyomas as a window for underlying disease. Furthermore, this case highlights the difficulties associated with managing chronic pain, especially for multiple lesions where surgical excision might not be practical or feasible. If the patient has a localized, few number of piloleiomyomas, surgical excision is accepted. Unfortunately, this is not always the case. In more complex cases, medications such as nifedipine and nitroglycerin target smooth muscle fibers and promote relaxation. Discomfort can also be relieved through gabapentin, pregabalin, and duloxetine. These are used to modify nerve signaling and reduce pain. Botulinum toxin injections have been explored, but its clinical effectiveness presents with inconsistency [14]. 

Several previously reported cases share clinical similarities with the presentation described in this report, reinforcing the diagnostic patterns and therapeutic approaches in managing cutaneous leiomyomas. In one case, a 10-year-old female in Turkey presented with a painful lesion over the right scapular region, accompanied by multiple firm nodules on the lumbar area that developed over a two-month period. Histopathological examination following a punch biopsy confirmed the diagnosis of pilar leiomyoma, characterized by spindle cells with eosinophilic cytoplasm [4,14]. Another case involved a 41-year-old Kuwaiti woman who developed gradually increasing, painful brown papules on the left forearm. The pain intensified upon cold exposure, mirroring the temperature-sensitive nature of leiomyoma-associated discomfort seen in the present case. Histological evaluation revealed elongated, cigar-shaped smooth muscle nuclei, confirming the diagnosis of cutaneous leiomyoma [4,14]. A third report described a 26-year-old woman with a painful erythematous plaque on the left shoulder, initially misdiagnosed as a keloid. Subsequent punch biopsy revealed smooth muscle proliferation, leading to a revised diagnosis of leiomyoma, which was successfully treated with surgical excision [4,14]. While these cases demonstrate consistent histopathological features and symptomatology, the current case contributes uniquely by documenting a middle-aged male with multiple clustered leiomyomas on the shoulder, highlighting the implications of diagnostic delay due to gradual symptom progression. Moreover, it emphasizes a non-surgical, pain-focused management approach, which is particularly relevant in multifocal disease where excision is not feasible. This report therefore adds to the growing body of evidence advocating for individualized, multimodal management strategies and underscores the importance of considering Reed’s syndrome in the presence of multiple lesions.

## Conclusions

Cutaneous leiomyomas, although rare, should be a key consideration in the differential diagnosis of painful dermal nodules, particularly when symptoms are paroxysmal and exacerbated by cold or mechanical pressure. Their clinical similarity to other benign skin lesions such as dermatofibromas and neurofibromas makes histopathological confirmation critical for accurate diagnosis and appropriate management. The identification of multiple lesions should prompt clinicians to investigate for underlying syndromic associations, most notably Reed’s syndrome, an autosomal dominant disorder with significant implications due to its association with renal cell carcinoma. Management is often challenging, with pain being the most disabling symptom. Pharmacologic therapy, particularly with calcium channel blockers like nifedipine, may offer symptomatic relief, although chronic pain often persists, necessitating long-term follow-up and multidisciplinary care.
